# Synthesis and Characterization of Green Zinc Oxide Nanoparticles with Antiproliferative Effects through Apoptosis Induction and MicroRNA Modulation in Breast Cancer Cells

**DOI:** 10.1155/2020/8817110

**Published:** 2020-11-20

**Authors:** Amir Hossein Aalami, Mohammad Mesgari, Amirhossein Sahebkar

**Affiliations:** ^1^Department of Biology, Mashhad Branch, Islamic Azad University, Mashhad, Iran; ^2^Department of Biology, Faculty of Science, Ferdowsi University of Mashhad, Mashhad 9177948974, Iran; ^3^Biotechnology Research Center, Pharmaceutical Technology Institute, Mashhad University of Medical Sciences, Mashhad, Iran; ^4^Neurogenic Inflammation Research Center, Mashhad University of Medical Sciences, Mashhad, Iran; ^5^Halal Research Center of IRI, FDA, Tehran, Iran; ^6^School of Pharmacy, Mashhad University of Medical Sciences, Mashhad, Iran

## Abstract

Changes in the expression of microRNAs can affect cancer cells' viability and behavior and the impact on cancer treatment. In this study, the expression of miR-155-5p, miR-203a-3p, and miR-223-3p in the MCF7 cancer cell line was studied when exposed to ZnO nanoparticles synthesized through a green route. Mentioned ZnO-NPs were well characterized by UV-vis spectroscopy, DLS, XRD, FTIR, FE-SEM, EDX, zeta potential, and AFM analyses. Cellular studies were conducted using ZnO-NPs before miRNA investigations including MTT cytotoxicity test against MCF7, MDA-MB-231, and HFF cell lines. Moreover, apoptosis assays were performed using morphological analysis, fluorescent dyes, flow cytometry, and evaluation of caspase-3 and caspase-8 gene expression. Biological properties such as the antioxidant and antimicrobial activity of these novel ZnO-NPs were considered. MTT assays showed that the inhibitory concentration (IC_50_) of ZnO-NPs after 24 h was 11.16 *μ*g/mL, 60.08 *μ*g/mL, and 26.3 *μ*g/mL on MCF7, MDA-MB-231, and HFF cells, respectively. The qRT-PCR results showed reduced expression of miR-155-5p, miR-203a-3p, and miR-223-3p when the MCF7 cells were treated with the IC50 concentration of ZnO-NPs (11.16 *μ*g/mL). The antioxidant activity results showed EC_50_ values at 57.19 *μ*g/mL and 31.5 *μ*g/mL in DPPH and ABTS assays, respectively. The antimicrobial activity of ZnO-NPs was determined on Gram-negative and Gram-positive bacterial strains and fungi using MIC and MBC assays. These NPs had a significant effect in reducing the expression of microRNAs in breast cancer cells. Finally, ZnO-NPs exerted antioxidant and antimicrobial activities.

## 1. Introduction

Biomedical nanomaterials have recently welcomed more attention because of their conspicuous biological characteristics and biomedical utilization [1]. Nanoparticles are being synthesized to multiple exciting and novel properties, which help their exploitation in entirely unrelated fields, such as nanodiagnostics [2], nanomedicine [3], antifungal [4], antioxidant [5], luminescence [6], anticancer drug/gene delivery [7], and photocatalytic potential [8].

Zinc oxide nanoparticles (ZnO-NPs) are essential metal oxides due to their impressive properties and full applications in various fields [9]. Compared with other metal oxides, ZnO-NPs are simple, low-cost, nontoxic, biosafe, and biocompatible and have been used as cosmetics, displaying desirable biomedical purposes, such as drug delivery, antibacterial, anticancer, and diabetes treatment, wound healing, anti-inflammation, and bioimaging [10, 11]. With these highlights, ZnO-NPs have introduced more attention in biomedical utilization. Additionally, zinc oxide is suggested as a generally recognized as safe (GRAS) material by the FDA [7, 12]. Furthermore, it can also generate reactive oxygen species (ROS) under ultraviolet light radiation to damage bacterial cell membranes [13].

*Saponaria officinalis (S. officinalis)*, also known as soapwort, belongs to Caryophyllaceae's family [14]. The roots of the *Saponaria* plant contain carbohydrates, tryptophan glycoside: saponarozidy, saponarozid, A, D, etc. However, alkaloids, ascorbic acid, flavonoids, vitexin, saponarin, and saponaretin are found in the leaves [15].

Saponins have various biological activities, including antioxidant, immunostimulant, hepatoprotective, antiulcer, antidiarrheal, anticancer, antitumor, antiplatelet, antidiabetic, and antibiotic effects [16, 17].

MicroRNAs (miRNAs) are noncoding RNA sequences with an estimated length of 20–25 nucleotides that have been preserved among a wide variety of species [18, 19]. miRNAs can control a diversity of biological functions, including cell division, proliferation, apoptosis, invasion, metastasis, development, metabolism, and tumorigenesis [20]. These molecules regulate the gene expression posttranscriptionally mainly through binding to the 3′' untranslated region of their target mRNAs [21].

Several miRNAs were found to control the formation and progression of breast cancer tumors, thus acting as oncomiRs, tumor-suppressor miRs, or metastatic miRs such as miR-155, miR-203, and miR-223 [21–25].

An important recent finding concerning the role of miR-155 in breast cancer is its relationship with BRCA1 [26]. BRCA1, the breast cancer sensitivity gene, is involved in DNA damage repair and cell cycle progression. Modifications of BRCA1 are correlated with a high risk of improving breast cancer [26]. In NMuMG cells, Smad4, a key signaling molecule in the TGF-*β* pathway, can bind to the BIC promoter and enrich miR-155 expression levels, thereby augmenting the TGF*β*-EMT process [27].

Among the microRNAs, miR-223 overexpression was responsible for downmodulating STAT5, ITGA3, and NRAS expressions at the protein level. In line with the miR-223 role, there is evidence suggesting that integrins, especially ITGA3 and ITGB1, are essential mediators of the outside-in and inside-out signaling in cancer, and their depletion leads to reduced cell migration and metastasis [28]. On the contrary, NRAS is a known oncogene, constitutively active in breast cancer and other predicted targets of miR-223, such as PI3K members and regulators [29].

Among many miRNAs quantified, miR-203 was downregulated in metastatic cells compared with nonmetastatic breast cancer cells [23]. Gene expression analysis showed the downregulation of various genes associated with cell motility and adhesion, such as CD44, ROCK1, and PTK2 in miR-135- and miR-203-treated cells. miR-203 also had an essential role in the expression of Runx2 in breast cancer cells and affected their tumorigenic features, both *in vitro* and *in vivo* [23]. Significantly, reconstitution with Runx2 restored the migration capacity of cells delivered with miR-203 [23].

Given the multitude of prognostic, diagnostic, and therapeutic effects of miRs [30], we studied the effects of characterized ZnO nanoparticles, synthesized with the aqueous extract of *S. officinalis*, in altering the expression of miRs and related biological effects in breast cancer cells.

## 2. Materials and Methods

### 2.1. Biosynthesis of ZnO-NPs

Fresh *Saponaria officinalis* was collected from the Hezar Masjed Mountains (36°59′04.0″N, 59°21′21.4″E). The leaves were cleaned by washing many times with running water and dehydrated for ten days at 25°C. The lab experiments were applied by double distilled water (ddw). *Saponaria officinalis* leaves (10 g of the dried leaf) were prepared in 100 mL of ddw. Then, they were placed on the heater stirrer at 70°C for 30 min. The extract was filtered and stored at 4°C.

To prepare 50 mL of 1 mmol/L solution of zinc acetate dihydrate, 0.0097 g of Zn (CH_3_COO)_2_.2H_2_O (Merck, Germany, CAS number: 5970-45-6) was dissolved in deionized water. Following the solid is entirely dissolved, the solution was diluted to an ultimate volume with deionized water.

The leaf extract was added to 50 mL of zinc acetate dihydrate Zn (CH_3_COO)_2_.2H_2_O at 35°C for 3 h on the magnetic stirrer adjusted to pH 11 by slow addition of 1 M NaOH (Merck, Germany). The precipitated NPs were dried and stored for forthcoming studies.

### 2.2. Characterization

The biogenic ZnO-NPs were analyzed by DLS (Cordouan Vasco3, France), UV-2550 Spectrophotometer (Shimadzu 2600i, Japan), FE-SEM + EDX (TESCAN, MIRA3, Brno, Czech Republic), X-ray diffraction (GNR Analytical Instruments, Italy), Fourier-transform infrared spectroscopy (Thermo Nicolet Avatar 370, USA), zeta potential (CAD, Zeta Compact, France), and atomic force microscopy (JPK, Germany).

### 2.3. *In Vitro* Cytotoxicity Assay

#### 2.3.1. Cell Culture and Viability

The MCF7, MDA-MB-231, and HFF cell lines were purchased from Pasteur Institute (Tehran, Iran) and cultured in the RPMI-1640 medium (Biosera, France) supplemented with 10% fetal bovine serum (FBS, Biosera, France) and 1% antibiotic under 5% CO_2_ at 37°C.

*In vitro* cytotoxicity of ZnO-NPs was investigated by the MTT assay. Cells were seeded in flat-bottom 96-well plates (5 × 10^3^) and incubated overnight. After 24 h, various concentrations of ZnO-NPs (0, 7.8, 15.6, 31.2, 62.5, 125, 250, and 500 *μ*g/mL) were added to the wells and subsequently incubated at 37°C for 24, 48, and 72 h. At the end of the incubation period, 20 *μ*L MTT solution (5 mg/mL in the medium, Sigma-Aldrich) was added to the wells, and the plate was incubated for 4 h. The supernatant was replaced by DMSO (150 *μ*uL/well, Sigma-Aldrich), and the absorbance was measured at 545 nm. Trials were performed in triplicate and repeated three times. IC_50_ (half-maximal inhibitory concentration) is the sample's concentration, which inhibits 50% of the cells. IC_50_ was used to assess the cytotoxicity of ZnO-NPs on cells using the following formula:

(1) $$\begin{array}{c} \text{growth inhibition }=\frac{\text{OD control }-\text{ OD treated sample}}{\text{OD control}}\times 100. \end{array}$$

#### 2.3.2. Acridine Orange/Propidium Iodide Staining (AO/PI)

The sufficient cell numbers were cultured for 24 hours at 37°C in a humidified CO_2_ incubator. The aim group was treated with ZnO-NPs, while the control group was treated with 0.1% DMSO. The cells were separated using trypsin. Fluorescent microscopy was performed by adding AO/PI dye to the solution at an equal ratio (10 *μ*L). Viability was evaluated based on the absorption of dye by the cells. The AO dye is only attached to the double-stranded DNA of viable and early apoptotic cells, while necrotic and dead cells only absorb PI dye.

#### 2.3.3. Hoechst Staining

Hoechst is generally accepted as staining that concurrently shows DNA and other cellular formations or proteins [31]. Besides, new reports have reported that exposure of Hoechst 33258 and Hoechst 33342 to UV light results in bleaching and photoconversion to sort with excitation/emission in the blue/green and green/red range. In this trial, MCF7 and HFF cells were administered with ZnO-NPs for 24 h. Then, the cells were collected and spread on clean slides. After that, the slides were air-dried, fixed in methanol and acetone (3 : 1, v/v), dyed with Hoechst 33258 (0.5 g/mL) for 20 min at 37°C, and then washed to remove unbound dye. Nuclear morphology was analyzed by fluorescence microscopy (Nikon 80i Eclipse, Japan) to recognize cell apoptosis.

#### 2.3.4. Cell Cycle Experiments

Flow cytometry was used to examine the kinetic cell cycle and apoptotic cell cultures [32]. For this purpose, around 5 × 10^3^ cells were grown after 24 h. Next, the cells were presented to different concentrations of ZnO-NPs in the RPMI medium. Following 24 h, drifting and attached cells were obtained, cleaned by PBS, then conveyed to divide microtubes, and dyed by 650 *μ*L PI liquid and incubated at the darkness for 15 min. Ultimately, the cell death cycle was confirmed by the cytometer device (Becton Dickinson, USA).

#### 2.3.5. qRT-PCR for mRNA and miRNA Detection

The expression of caspase-3 and caspase-8 as apoptotic genes and three microRNAs as a biomarker were calculated by qRT-PCR with peculiar primers. *β*-Actin and GAPDH were used as the normalized gene for caspases, and small nucleolar RNA (SNORD 47) was used as a housekeeping gene for miRNAs (Table 1).

Total RNA was obtained utilizing Norgen's Total RNA Purification Kit (Ontario, Canada). Then, complementary DNAs (cDNAs) were generated with the TruScript First Strand cDNA Synthesis Kit (Ontario, Canada) and BON-miR cDNA Synthesis Kit (Bonyakhteh, Iran). Real-time qPCR was performed by ABI device Step one (Applied Biosystems, USA).

### 2.4. Antioxidant Activity

#### 2.4.1. DPPH Radical Scavenging Activity

Antioxidant activity was performed using a modified 2, 2-diphenyl-1-picrylhydrazyl (DPPH) assay according to the method described by Brand-Williams et al. [33]. 39.4 mg of DPPH (Sigma-Aldrich, USA) was dissolved in 100 mL of ethanol 96% to prepare DPPH 0.1 mM stock and stored in the refrigerator (2–8°C) for further assessments. On the contrary, 3 mg of NPs was added to 3 mL ddw to achieve 1 mg/mL concentration. Serial dilution was performed with concentrations of ZnO (0–500 *μ*g/mL) and mixed with 500 *μ*L of 1 mM DPPH solution. The combination was incubated at room temperature in darkness about 30 min. Butylated hydroxyanisole (BHA), a powerful antioxidant, was considered as the positive control, and ethanol was considered as the negative control. The absorbance was read at 517 nm. The % inhibition scavenging activity was assessed as follows:

(2) $$\begin{array}{c} \%\text{ antioxidant activity }=\frac{A_{c}-A_{\text{s}}}{A_{\text{c}}}\times 100, \end{array}$$

where *A*_*c*_ and *A*_s_ are the absorbance of the control and sample, respectively.

#### 2.4.2. ABTS^+^ Radical Scavenging Activity

ABTS^+^ (2, 2-azino-bis (3-ethylbenzothiazoline-6-sulfonic acid)) solution was prepared by the instruction of Giao et al. [34]. ABTS^+^ (Biobasic, Canada) was mixed with potassium persulfate (Sigma-Aldrich, USA) in distilled water and incubated for a day at 25°C in darkness. ABTS^+^ decolorization assay requires the production of the ABTS^+^ chromophore through the oxidation of ABTS^+^ by potassium persulfate [35]. The inhibition of the ZnO-NPs on ABTS^+^ radicals was estimated at 734 nm after 30 min. The % inhibition was assessed as follows:

(3) $$\begin{array}{c} \%\text{ antioxidant activity }=\frac{A_{c}-A_{\text{s}}}{A_{\text{c}}}\times 100, \end{array}$$

where *A*_*c*_ and *A*_s_ are the absorbance of the control and sample, respectively.

### 2.5. Antimicrobial Activity

The antimicrobial activity of different concentrations of ZnO-NPs (0–1250 *μ*g/mL) was evaluated against ATCC strains *P. aeruginosa*, *E. coli*, *B. subtilis*, *S. aureus*, *A. baumannii*, and *C. albicans* applying a broth microdilution plan according to the CLSI guidelines [36]. Minimum inhibitory concentration (MIC) and minimum bactericidal concentration (MBC) were measured and reported as described previously [37].

### 2.6. Statistical Analysis

Data were analyzed using Statistical Package for Social Sciences (SPSS) software version 21 (IBM Inc., Chicago, IL, USA). All tests were performed three times, and the results were presented as mean ± standard deviation (SD). Graphs were created using GraphPad Prism 8.0 (GraphPad Software Inc., CA, USA). The normalized genes for each sample were calculated by the Livak method (2^−ΔΔCt^). To calculate the difference in the gene expression between groups, GenEX software (Exiqon AS/MultiD Analyses) version 6.0 was used. One-way ANOVA statistically analyzed multiple comparisons with a Dunn–Sidak post hoc test. The level of statistical significance was accepted as *p* < 0.05.

## 3. Results

### 3.1. ZnO-NPs' Characterization

#### 3.1.1. UV-Vis Spectral Analysis

**Figure 1:**
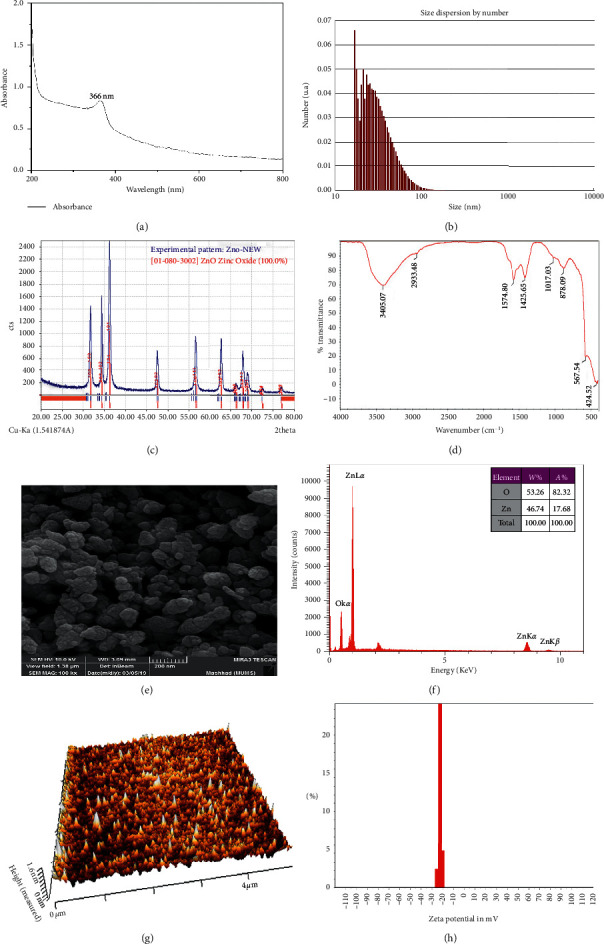
The green synthesized ZnO-NP characterization data. (a) The UV-vis spectra of the reaction mixture containing *S. officinalis*-mediated ZnO nanoparticles (ZnO-NPs). (b) Size distribution of *S. officinalis* ZnO-NPs as analyzed by DLS. The result showed that the nanoparticles are well dispersed, and the nanoparticles' average size is 31.5 nm. (c) The XRD pattern of *S. officinalis* ZnO-NPs. The nanoparticles' XRD pattern revealed distinct peaks at 31.73°, 34.39°, 36.22°, 47.51°, 56.56°, 62.81°, 66.29°, 67.91°, and 68.98°. Consequently, the XRD pattern confirmed that the ZnO-NPs have a crystalline structure. (d) FTIR of *S. officinalis* ZnO-NPs. The peaks recognized in the nanoparticle solution describe the operative groups of the chemical compounds of *S. officinalis* that decreased zinc to produce the nanoparticles. (e) The field emission scanning electron microscope (FE-SEM) view of *S. officinalis* ZnO-NPs. The formed ZnO-NPs were observed with a scanning electron microscope to identify their forms and sizes. Based on the microscopic image, the nanoparticles have a spherical shape. (f) Energy-dispersive X-ray (EDX) spectra of *S. officinalis* ZnO-NPs. The absorption peak found around the 1 keV shows zinc oxide nanoparticles' configuration and appearance in the reaction mixture. (g) Atomic force microscopy (AFM) of *S. officinalis* ZnO-NPs. The image of AFM shows that the synthesized ZnO nanoparticle diameter was 1.6 nm. (h) Zeta potential graph of *S. officinalis* ZnO-NPs. The zeta potential evaluated the stability of the synthesized ZnO nanoparticles. The zeta potential of the nanoparticles was found to be −23.5 mV, which indicates the nanoparticles are almost stable.

The white precipitate was dehydrated in the oven to produce ZnO nanoparticles. The powder was added in deionized water to record an absorption peak at 366 nm for ZnO-NPs (Figure 1(a)). Previous research has described that ZnO-NPs give an absorption peak between 340 and 380 nm, revealing surface plasmon resonance (SPR) [5, 38].

#### 3.1.2. DLS Measurement

The dynamic light scattering device is known to estimate the thickness of metallic nanoparticles (MNPs). The average size of ZnO-NPs was estimated by DLS measurement, and the distribution vs. intensity graph has been displayed in Figure 1(b). The average size of synthesized ZnO-NPs was 31.5 nm.

#### 3.1.3. XRD Analysis

X-ray diffraction pattern peaks for ZnO-NPs were observed with 2*θ* at 31.73° (1 0 0), 34.39° (0 0 2), 36.22° (1 0 1), 47.51° (1 0 2), 56.56° (1 1 0), 62.81° (1 0 3), 66.29° (2 0 0), 67.91° (1 1 2), and 68.98° (2 0 1). Therefore, the XRD pattern matches with the stipulations stated by the Joint Committee on Powder Diffraction Standard (JPDS) (file no: 36-1451) for metallic zinc [39].

Crystalline size was estimated from the full peak corresponding to the (1 0 1) plane by applying the Debye–Scherrer formula [40].

Scherrer's formula is *D* = 0.89*λ*/*β*cos*θ*, where 0.89 = Scherrer's constant, *λ*  = X-ray wavelength (1.5418 Å), *β* = FWHM (full width at half maximum), and *θ* = Bragg's angle of diffraction. The crystallite size for (1 0 0), (0 0 2), (1 0 1), (1 0 2), (1 1 0), (1 0 3), (2 0 0), (1 1 2), and (2 0 1) peaks was found to be 18.58, 18.71, 18.80, 19.52, 20.29, 20.94, 21.34, 21.54, and 21.68 nm, respectively, and the average of crystallite size was calculated as 20.16 nm (Figure 1(c)).

#### 3.1.4. FTIR Spectral Analysis

The FTIR spectra appeared in several peaks at 3405.07, 2933.48, 1574.8, 1425.65, 1017.03, 878.09, 561.54, and 424.52 cm^−1^ (Figure 1(d)). The peaks at 3405.07 cm^−1^ match to O-H of water [41]. The peak at 2933.48 cm^−1^ refers to vibrations of the C-H stretch of alkynes [42]. The peaks at 1574.8 cm^−1^ refer to C=O functional groups [42]. The peak at 1425.65 cm^−1^ indicates to amine -NH in protein amide linkages [2]. The peak at 1017.03 cm^−1^ results from the C-N stretch of aliphatic amines [2]. The peak at 878.09 cm^−1^ corresponds to aromatic C-H bonding. The peaks in the range of 400–600 cm^−1^ are assigned to ZnO [42].

#### 3.1.5. FE-SEM and EDX

The FE-SEM image of ZnO is shown in Figure 1(e). The diameter of the ZnO-NPs was determined in the range of 20–80 nm with spherical form and rough cover. This image confirmed the value of XRD crystallite data. Compared to our study, the morphology of the biosynthesized ZnO-NPs was presented by Sujatha et al., with similar shapes and sizes ranging between 80 and 130 nm [43].

EDX analysis confirmed O and Zn elements' attendance on the surface of the sample (Figure 1(f)). Vanathi et al. reported similar peaks [44].

#### 3.1.6. Atomic Force Microscope Analysis

Atomic force microscopy (AFM) was employed to recognize the sample's external morphology and roughness. The diameters of a hundred random particles were estimated on the *Z*-axis (height). The determined diameter of the synthesized ZnO nanoparticle was 1.6 nm. It can be observed entirely in Figure 1(g) that the 3D image of ZnO-NP morphology has been spherical.

#### 3.1.7. Zeta Potential Measurement

Zeta potential (ZP) provides information as to the surface charge and stability of ZnO-NPs. In this study, there was a small difference in the zeta potential of ZnO-NPs. In general, the consequences of zeta potential peaks for ZnO-NPs at pH about 7 were around −23.5 mV, which showed that it is constant due to the electrostatic repulsive force (Figure 1(h)).

### 3.2. Anticancer Studies

#### 3.2.1. Cell Viability

**Figure 2:**
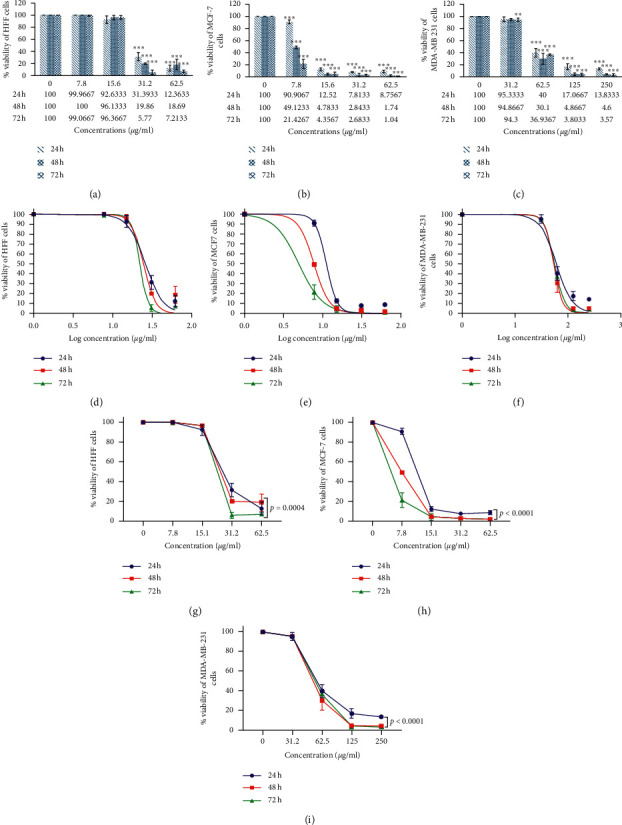
MTT assay analysis. Results were presented as mean ± SD (*n* = 3). The IC_50_ value was calculated as 11.16 *μ*g/mL, 26.3 *μ*g/mL, and 60.08 *μ*g/mL for MCF7, HFF, and MDA-MB-231, respectively, after 24 h incubation with ZnO-NPs. The diagrams were performed by Tukey comparison and showed the antiproliferative capacity of ZnO-NPs against HFF (a), MCF7 (b), and MDA-MB-231 (c) cell lines after 24, 48, and 72 hours, respectively. The nonlinear regression sigmoidal dose-response and logarithmic transformation of ZnO concentration showed the logarithmic concentrations tested on cell lines after 24, 48, and 72 hours (d–f). Two-way ANOVA showed a significant level between different times and concentrations of MCF7, HFF, and MDA-MB-231 cell lines (g–i).

Cytotoxicity increased by increasing the concentration of ZnO nanoparticles. There was a modification in the rate of cell viability in the control. The MCF7, MDA-MB-231, and HFF cells were treated by ZnO-NPs (0–500 *μ*g/mL). IC_50_ of biogenic ZnO-NPs was calculated as 11.16 *μ*g/mL, 26.3 *μ*g/mL, and 60.08 *μ*g/mL for MCF7, HFF, and MDA-MB-231, respectively, following 24 h incubation (Figure 2). Due to the high resistance of the MDA-MB-231 cell line to nanoparticles compared to the control group, it was excluded from the experiments (Table 2).

#### 3.2.2. Determination of Cytomorphological Modifications on Cell Lines

**Figure 3:**
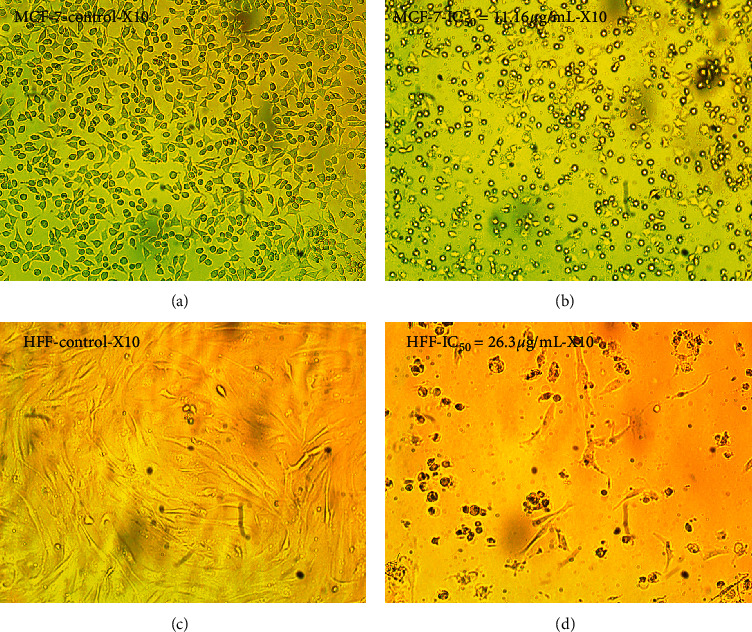
Morphological alteration of cancer cells exposed to the IC_50_ (24 h) concentration of ZnO-NPs compared with the control. (a) Untreated MCF7 cells. (b) Morphology of the MCF7 cells after ZnO-NP treatment with IC_50_ dose (IC_50_ = 11.16 *μ*g/mL). (c) Untreated HFF cell morphology. (d) HFF cells' morphology after ZnO-NP treatment with IC_50_ dose (IC_50_ = 26.3 *μ*g/mL).

Various morphological modifications were recognized in ZnO-NP-treated MCF7 and HFF cells; however, such effects were not remarked in untreated cells. Morphological changes were observed, including the destruction of membrane integrity, cell growth inhibition, cytoplasmic density, and cell retraction (Figure 3). The results also confirmed the mortality of MCF7 and HFF cells affected by ZnO-NPs, whereas the untreated cells were active.

### 3.3. Apoptosis Assays

#### 3.3.1. AO/PI Morphological Changes

**Figure 4:**
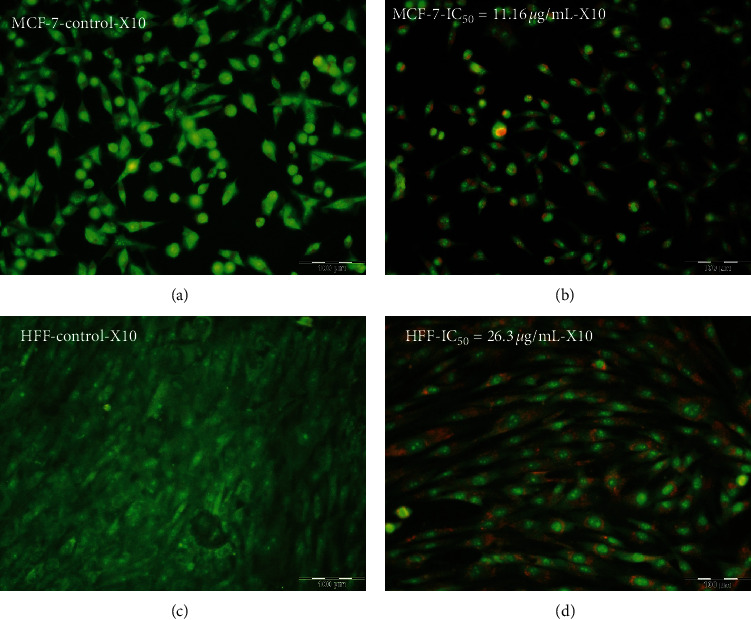
Effect of zinc oxide nanoparticles on apoptotic morphological changes in MCF7 and HFF cell lines by AO/PI staining. (a) Untreated MCF7 cells. (b) Morphology of the MCF7 cells after ZnO-NP treatment with IC_50_ dose (IC_50_ = 11.16 *μ*g/mL). (c) Untreated HFF cell morphology. (d) HFF cells' morphology after ZnO-NP treatment with IC_50_ dose (IC_50_ = 26.3 *μ*g/mL).

The apoptosis has been known by the morphological differences in the cell form and chromatin reduction. The stained cells were detectable as viable (light green), as initial apoptotic, late apoptotic (orange fluorescence), and nonviable cells (red-colored fluorescence) (Figure 4). Furthermore, different nuclear morphologies, including condensed nuclei, blebbing, membrane, and apoptotic bodies, were recognized in ZnO-NP-treated MCF7 and HFF cells.

#### 3.3.2. Hoechst Staining

**Figure 5:**
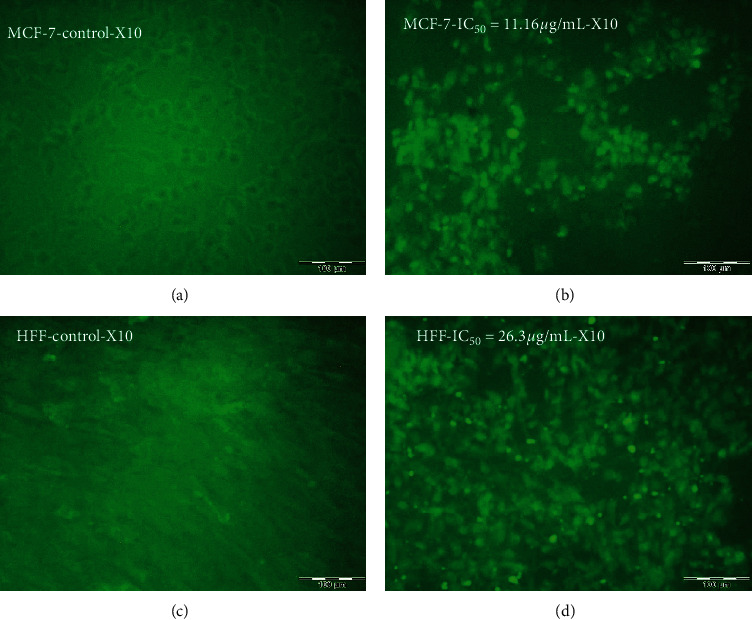
Effect of zinc oxide nanoparticles (ZnO-NPs) on apoptotic morphological changes in MCF7 and HFF cell lines by Hoechst staining. (a) Untreated MCF7 cells. (b) Morphology of the MCF7 cells after ZnO-NP treatment with IC_50_ dose (IC_50_ = 11.16 *μ*g/mL). (c) Untreated HFF cell morphology. (d) HFF cells' morphology after ZnO-NP treatment with IC_50_ dose (IC_50_ = 26.3 *μ*g/mL).

In Hoechst staining, following treatment MCF7 with 11.16 *μ*g/mL and HFF with 26.3 *μ*g/mL of ZnO-NPs for 24 h, apoptotic signs such as cellular shrinkage, nuclear consolidation, and fragmentation were noticeable. In the control group, the cells were regular in shape and revealed a natural nuclear structure (Figure 5). Such morphological changes suggest activation of caspase cascades.

#### 3.3.3. Cell Cycle Phase Distribution

**Figure 6:**
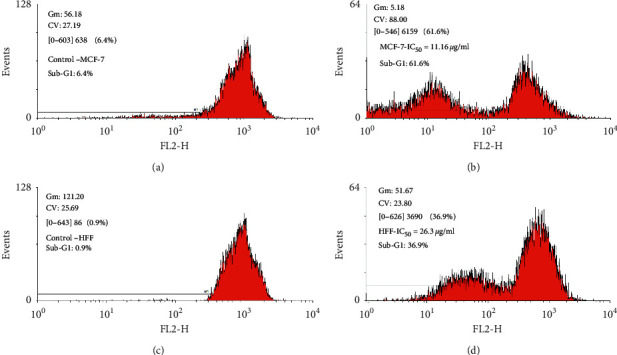
The effect of *S. officinalis*-mediated ZnO-NPs on the cell cycle progression in MCF7 and HFF cell lines. The cell cycle analysis of MCF7 and HFF cells after 24 h of treatment with 11.16 *μ*g/mL and 26.3 *μ*g/mL of ZnO-NPs, respectively, revealing that the population of cells in the sub-G1 phase increased with increasing ZnO-NPs in IC_50_ compared with untreated cells.

The outcomes of flow cytometry revealed an increase in sub-G1 cells treated with ZnO-NPs compared to the control. The population of sub-G1 cells in the MCF7 cells exposed to 11.16 *μ*g/mL ZnO-NPs showed an apoptotic cell increase by 61.6%, while in the control group, it was 6.4%. Similar differences between the percentage of the sub-G1 population in HFF cells treated with 26.3 *μ*g/mL ZnO-NPs (36.9%) and nontreated (0.9%) was seen. The increase of ZnO-NP doses leads to the increment of sub-G1 peak values. The sub-G1 peak value at the IC_50_ concentration of ZnO-NPs confirmed the apoptotic sort of death (Figure 6).

#### 3.3.4. Caspase-3 and Caspase-8 Gene Expression

**Figure 7:**
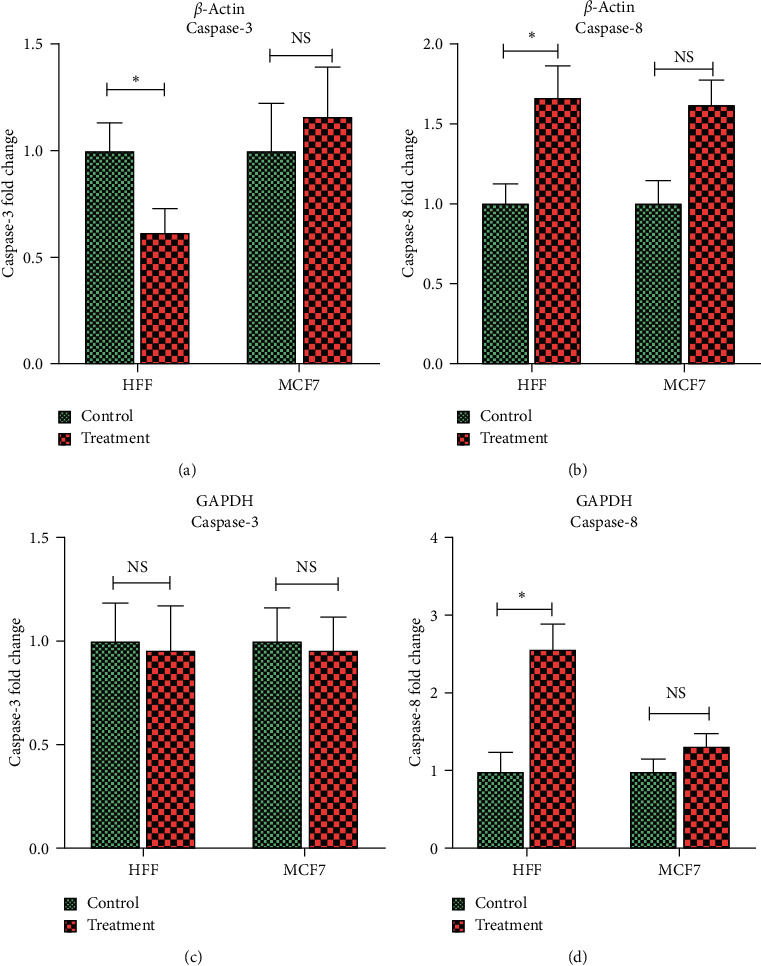
Caspase-3 and Caspase-8 gene expression in MCF7 breast cancer and HFF cells after 24 h incubation with their IC_50_ dose of ZnO-NP concentrations. The Tukey comparison of the fold change values was performed for the caspase-3 and caspase‐8 gene expression in ZnO-NP-treated and untreated cells based on *β*-actin and GAPDH internal controls. In the diagram analyses, the control group was used as a reference for calculating fold change. ∗ refers to the significance, *p* < 0.05.

 Caspase-3 and caspase-8 have a direct role in the apoptosis process. Real-time quantitative PCR was performed to examine the impact of ZnO-NPs on the caspase-3 and caspase-8 gene expression in MCF7 and HFF cells. Results showed that the expression level of these genes was different between ZnO-NP-treated (at IC50 concentration) versus nontreated cells in both HFF-1 and MCF7 cells, *p* < 0.05 (Figure 7) (Table 3).

#### 3.3.5. MicroRNA Expression

The expression of miR-155-5p in HFF cells with treatment was 2.15 ± 0.32 times more than the control group (*p* < 0.015). However, miR-155-5p expression was significantly downregulated in MCF7 treatment cell lines compared to the control (*p* = 0.016).

**Figure 8:**
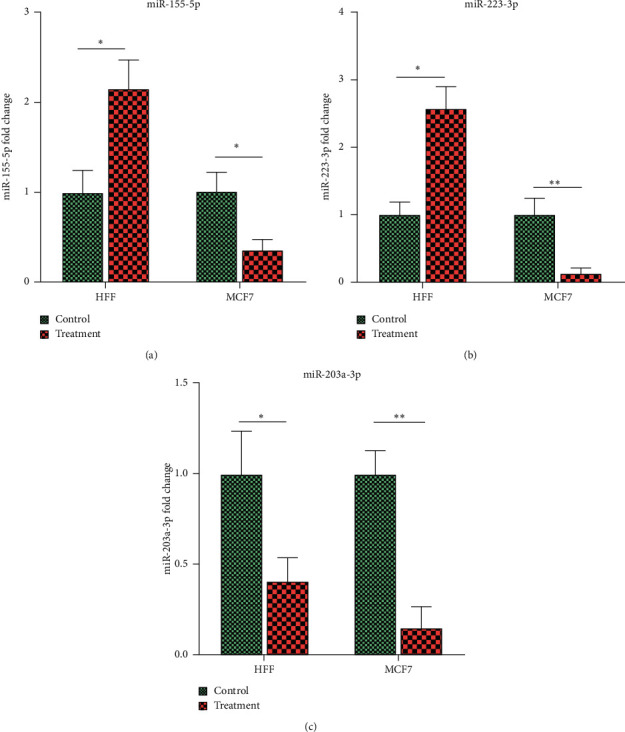
The Tukey multiple comparisons showed the *x*-level expression of miR-155-5p (a), miR-223-3p (b), and miR-203a-3p (c) between treatment and control groups in HFF and MCF7 cell lines. In these diagrams, the control group was used as the reference group for each cell line. Each cell line was analyzed separately, and only their diagrams were displayed side by side. In other words, it is not possible to compare different cell lines; for example, the HFF control group cannot be compared with the MCF7 control group. ∗ refers to the significance *p* < 0.05, and ∗∗ refers to *p* < 0.01.

The expression of miR-223-3p in HFF cells with treatment was 2.57 ± 0.32 times more than the nontreatment group (*p* < 0.011). However, in MCF7, miR-223-3p, like miR-155-5p, was downregulated after treatment with ZnO-NPs. There was a meaningful difference in the miR-203a-3p expression between treatment and control groups in HFF and MCF7 cell lines (*p* < 0.025 and *p* < 0.08, respectively) (Table 4). The miR-203a-3p expression was 0.40 ± 0.32 and 0.14 ± 0.12 in HFF and MCF7 cells after treatment, respectively (Figure 8).

### 3.4. Antioxidant Activity

#### 3.4.1. DPPH Radical Scavenging Activity

**Figure 9:**
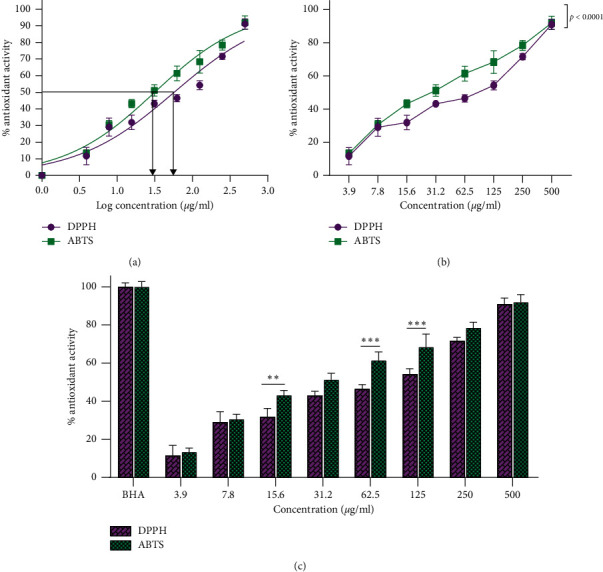
Inhibiting effect of ZnO-NPs on DPPH and ABTS radicals. (a) The nonlinear regression sigmoidal dose-response and logarithmic transformation of the ZnO-NP concentrations showed that EC_50_ for ABTS and DPPH is log 1.498 and log 1.757, respectively. (b) Two-way ANOVA showed the significant difference between ABTS and DPPH tests at different concentrations of ZnO-NPs. (c) Dunn–Šidák comparison test indicated a significant difference compared to DPPH and ABTS radicals at the same concentration. ^*∗∗∗*^*p* < 0.001; ^*∗∗*^*p* < 0.01.

The DPPH assay was employed to evaluate the antioxidant activity of ZnO-NPs. Free radical scavenging activity was measured by reading the absorbance at 517 nm by using the UV-vis spectrophotometer. Results showed that the zinc oxide nanoparticles restrained the free radicals in contrast to the control group (Figures 9(a) and 9(b)). EC_50_ was determined at 57.19 *μ*g/mL ZnO-NPs (Table 5), while at the same concentration of the standard sample displayed 100% activity inhibition. The maximum scavenging activity of ZnO-NPs was observed at 500 *μ*g/mL, with 91.07% inhibition.

#### 3.4.2. ABTS Radical Scavenging Activity

The free radical scavenging effect in ABTS increased with the rising concentration of ZnO-NPs. Figures 9(a) and 9(b) confirmed the ZnO-NP inhibition effect on ABTS free radicals. EC_50_ was calculated at 31.5 *μ*g/mL (Table 5). The maximum scavenging activity of ABTS was observed at 91.91% in 500 *μ*g/mL of ZnO-NPs. Overall, the free radical scavenging result was raised with the rising intensity of ZnO-NPs. Comparison of the amount of free radical scavenging at different concentrations of ABTS and DPPH is shown in Figure 9(c).

### 3.5. Antimicrobial Activity

**Figure 10:**
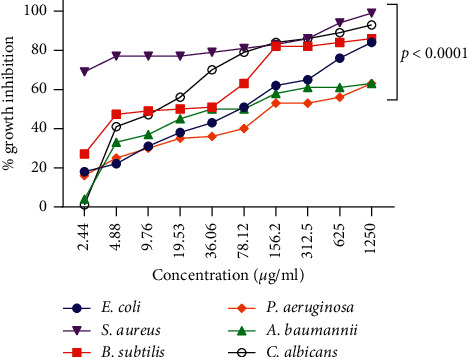
The growth inhibition happened by *S. officinalis*-mediated ZnO-NP concentrations against Gram-positive strains (*B. subtilis* and *S. aureus*), Gram-negative strains (*A. baumannii, E. coli,* and *P. aeruginosa*), and *C. albicans*. This diagram showed that the *S. officinalis*-synthesized ZnO nanoparticles showed a powerful antibacterial effect for *S. aureus* and a weaker effect on *P. aeruginosa*.

According to the current articles, green ZnO-NPs were discovered to possess excellent antibacterial and antifungal activity [45]. The MIC and MBC of our ZnO-NPs (with the average particle size of 20–40 nm) against ATCC strains *P. aeruginosa*, *E. coli*, *B. subtilis*, *S. aureus*, *A. baumannii*, and *C. albicans* were measured (Figure 10). More than 50% of both bacterial cell walls were lost (Table 6). Subculturing MIC wells also determined the MBC values in the nutrient agar medium. Therefore, nanoparticles can be applied as a potential antimicrobial factor and support to overcome the complications in pathogenesis bacteria control posed by the improvement of resistance to conventional bactericide.

## 4. Discussion

One of the numerous known characteristics of cancer cells is their ability to evade apoptosis, which allows unlimited and invasive proliferation [46]. The 31.5 nm ZnO-NPs were synthesized utilizing a green synthesis method using the *S. officinalis* aqueous leaf extract. The viability, morphology, and apoptotic properties of cancer cells were assessed after treatment with ZnO-NPs. Our findings confirmed the significant positive correlation between the apoptotic death and nontoxic concentrations of ZnO-NPs. Many studies have demonstrated the cytotoxic effect of various metal nanoparticles, including ZnO-NPs, on cancer cells.

Anitha et al. [45] reported that IC_50_ of ZnO-NPs from *Artocarpus gomezianus* fruits was 9.34 *μ*g/mL after 24 h in the MCF7 cell line. They also confirmed the antibacterial and antifungal activity of ZnO-NPs against *Staphylococcus aureus* and *Aspergillus niger*.

Hackenberg et al. [47] showed the toxicity of ZnO-NPs on nasal mucosal cells evaluated by MTT and trypan blue assays, in which they reported cytotoxicity at 50 *μ*g/mL concentration. Mahdizadeh et al. [48] determined that IC_50_ of ZnO-NPs from *Cucumis melo* was 40 *μ*g/mL in MCF7 and 20 *μ*g/mL in TUBO cells after 24 h of incubation. Umar et al. [49] showed that ZnO-NPs from *Albizia lebbeck* have antimicrobial, antioxidant, and cytotoxic activities. They reported that the 100 *μ*g/mL concentration of ZnO-NPs inhibits the cell number of MDA-MB-231 and MCF7 by 76.8% and 80.2%, respectively. Hanley et al. [50] showed the cytotoxic effects of ZnO-NPs on normal and malignant breast, epithelial, and prostate cell lines, as well as on T and B lymphocytes. Normal T and B lymphocytes showed maximum resistance to the toxicity of ZnO-NPs. It was found that particle charge can affect cytotoxicity induction because cationic NPs were more toxic than neutral and anionic particles, which could be applied in cancer treatment.

In this study, cytotoxicity induced on MCF7, MDA-MB-231, and HFF was evaluated following treatment with ZnO-NPs synthesized from the leaf extract of *S. officinalis*, which was assayed at 24, 48, and 72 hours. The results showed the remarkable inhibitory effects of NPs on the growth of breast cancer cells. Compared to the control group, *S. officinalis* ZnO-NPs decreased the size and number of cells and resulted in cell deformation. It was found that, with increasing concentration, the cytotoxic effect of ZnO-NPs on cancer cells was increased.

Alarifi and colleagues explored the cytotoxic, oxidative, and proapoptotic effects of ZnO-NPs in A375 human skin melanoma cells. The results showed the induction of apoptosis as confirmed by chromosome densification and caspase-3 activation tests. Also, more considerable DNA damage was observed in cells treated with the highest concentration of ZnO-NPs. These results indicate that ZnO-NPs have genotoxic potential in A375 cells and may induce oxidative stress. Short-term investigations of the induction of genotoxicity and apoptotic responses to ZnO-NPs require further investigations to determine whether there may be long-term consequences of exposure to ZnO-NPs [51].

In a study on human keratinocytes, ZnO-NPs were found to augment reactive oxygen species (ROS) production and initiate oxidative stress. The results of this study indicate that ZnO-NPs have the potential to stimulate ROS production. Hence, the mechanism of nanotoxicity is secondary to ROS production, oxidative stress, and apoptosis [52]. Various therapeutic strategies have been used for targeting the Cas‐3 protein in breast cancer cells [53]. Studies have confirmed that ZnO-NPs have proapoptotic properties. In the present study, expression analysis of genes involved in the apoptosis process (caspase-3 and caspase-8) indicated that ZnO-NPs prepared using the leaves of *S. officinalis* had proapoptotic effects compared to control cells because at a concentration of 11.16 *μ*g/mL, 50% of MCF7 cells showed programmed cell death, as well as induction of caspase-8 expression. To the best of our knowledge, this is the first study assessing the impact of green synthesized ZnO-NPs on the expression of apoptosis-effector genes, Cas‐3 and Cas‐8, in breast cancer cells (MCF7) and human foreskin fibroblasts (HFF). Mahdizadeh et al. [48] reported that ZnO-NPs from *Cucumis melo inodorus* blunted cancer cell growth via inducing the activity of caspases and triggering apoptosis. Their data suggested that ZnO-NPs can increase the number of cells in the sub-G1 phase and induce DNA destruction and apoptosis, consistent with the present results. Bai et al. [54] showed the proapoptotic effects of ZnO-NPs by inducing ROS production and ensuing oxidative stress in human ovarian cancer cells (SKOV3).

Oxidative stress is caused by the activation of caspases and can culminate in apoptosis induction. Endonova et al. [15] showed that the leaves of *S. officinalis* have the highest antioxidant content of flavonoids (53.39 mg/g) compared to roots and blossom clusters. We compared ZnO-NPs with BHA in terms of their antioxidant capacities. Our results showed a significant antioxidant capability (91.91%) for ZnO-NPs at 500 *μ*g/mL concentration for ABTS and 91.07% for DPPH (Figure 9).

ZnO-NPs have antifungal and antibacterial properties [55, 56]. They have biological effects on microorganisms such as *S. pyogenes, B. cereus, E. coli*, and *P. aeruginosa*, in which they increase the permeability of the bacterial cell membrane [57]. In this regard, our research demonstrated that ZnO-NPs have antimicrobial effects against pathogenic bacteria such as *E. coli*, *P. aeruginosa*, *S. aureus*, *B. subtilis, A. baumannii,* and *C. albicans*. The findings of this study, for the first time, presented the use of miR-155-5p, miR-223-3p, and miR-203a-3p as potential effectors of ZnO-NPs—synthesized using a green method—in HFF and MCF7 cell lines. In summary, the expression levels of miR-155-5p, miR-223-3p, and miR-203a-3p were downregulated after treatment with ZnO-NPs.

## 5. Conclusion

The present study showed an eco-friendly synthesis of ZnO-NPs using the aqueous leaf extract of *S. officinalis* for the first time. Synthesized NPs exhibited a spherical shape with a size range of 20–40 nm. The synthesized ZnO-NPs showed potential free radical scavenging capability, which was confirmed by DPPH and ABTS assays. The green synthesized ZnO-NPs demonstrated inhibitory effects against pathogenic bacteria. Moreover, ZnO-NPs efficiently inhibited MCF7 cancer cells' growth under the *in vitro* condition and downregulated the expression of miRs. Given the present findings, the synthesized ZnO-NPs could have potential biomedical and pharmaceutical applications.

## Figures and Tables

**Table 1:** The sequences of forward and reverse designed primers for target genes.

Gene name	Primers (5′ ⟶ 3′)	Accession number
Caspase-3	Forward: CTGGACTGTGGCATTGAGAC	NM_001224
	Reverse: GCAAAGGGACTGGATGAACC	

Caspase-8	Forward: GAAAAGCAAACCTCGGGGATAC	NM_001228
	Reverse: CCAAGTGTGTTCCATTCCTGTC	

*β*-Actin	Forward: TGAGAGGGAAATCGTGCGTG	NM_205518
	Reverse: GTTGGCATAGAGGTCTTTACGG	

GAPDH	Forward: GCCTTCCGTGTTCCTACCC	NM_002046
	Reverse: TGCCTGCTTCACCACCTTC	

hsa-miR-155-5p	Forward: UUAAUGCUAAUCGUGAUAGGGGUU	MIMAT0000646
hsa-miR-203a-3p	Forward: GUGAAAUGUUUAGGACCACUAG	MIMAT0000264
hsa-miR-223-3p	Forward: UGUCAGUUUGUCAAAUACCCCA	MIMAT0000280
SNORD47	Forward: CGCCAATGATGTAATGATTCTG	
Universal reverse primer	Universal reverse primer was obtained from Bonyakhteh Company (Bonyakhteh, Tehran, Iran)	

Abbreviations: miR: microRNA, GAPDH: glyceraldehyde 3‐phosphate dehydrogenase, and SNORD47: small nucleolar RNA.

**Table 2:** The nonlinear regression sigmoidal dose-response curve calculated the different IC_50_ values of ZnO-NPs for the breast cancer cell lines (MCF7 and MDA-MB-231) and HFF.

		MCF7	MDA-MB-231	HFF
IC_50_	24 h	11.16 *μ*g/mL	60.08 *μ*g/mL	26.3 *μ*g/mL
	48 h	7.736 *μ*g/mL	53.28 *μ*g/mL	24.76 *μ*g/mL
	72 h	4.941 *μ*g/mL	55.83 *μ*g/mL	22.34 *μ*g/mL

Log IC_50_	24 h	1.048 *μ*g/mL	1.779 *μ*g/mL	1.42 *μ*g/mL
	48 h	0.8885 *μ*g/mL	1.727 *μ*g/mL	1.394 *μ*g/mL
	72 h	0.6938 *μ*g/mL	1.747 *μ*g/mL	1.349 *μ*g/mL

Abbreviations: MCF7: Michigan Cancer Foundation-7, HFF: human foreskin fibroblasts, IC_50_: the half-maximal inhibitory concentration, and log: logarithm.

**Table 3:** The *t*-test was performed for the fold change values for caspase-3 and caspase-8 gene expression analysis in ZnO-NP-treated cells.

Variable		Caspase-3 expression (fold change) based on *β*-actin	*p* value	Caspase-3 expression (fold change) based on GAPDH	*p* value

HFF	Control	1.00 ± 0.13	0.020	1.00 ± 0.18	0.819
	Treatment	0.61 ± 0.11		0.952 ± 0.21	

MCF7	Control	1.00 ± 0.22	0.239	1.00 ± 0.16	0.821
	Treatment	1.16 ± 0.23		0.959 ± 0.15	

Variable		Caspase-8 expression (fold change) based on *β*-actin	*p* value	Caspase-8 expression (fold change) based on GAPDH	*p* value

HFF	Control	1.00 ± 0.12	0.020	1.00 ± 0.24	0.016
	Treatment	1.65 ± 0.20		2.56 ± 0.32	

MCF7	Control	1.00 ± 0.14	0.1	1.00 ± 0.16	0.41
	Treatment	1.61 ± 0.15		1.33 ± 0.15	

Abbreviations: MCF7: Michigan Cancer Foundation-7, HFF: human foreskin fibroblasts, miR: microRNA, and GAPDH: glyceraldehyde 3‐phosphate dehydrogenase.

**Table 4:** The *t*-test was performed for the comparison of microRNA expression between control and treatment groups in the MCF7 and HFF cell lines.

Variable		miR-155-5p expression (fold change)	*p* value

HFF	Control	1.00 ± 0.24	0.015
	Treatment	2.15 ± 0.32	

MCF7	Control	1.00 ± 0.22	0.016
	Treatment	0.35 ± 0.32	

Variable		miR-223-3p expression (fold change)	*p* value

HFF	Control	1.00 ± 0.19	0.011
	Treatment	2.57 ± 0.32	

MCF7	Control	1.00 ± 0.25	0.008
	Treatment	0.12 ± 0.09	

Variable		miR-203-3p expression (fold change)	*p* value

HFF	Control	1.00 ± 0.24	0.025
	Treatment	0.40 ± 0.13	

MCF7	Control	1.00 ± 0.13	0.008
	Treatment	0.14 ± 0.12	

Abbreviations: MCF7: Michigan Cancer Foundation-7, HFF: human foreskin fibroblasts, and miR: microRNA.

**Table 5:** The nonlinear regression sigmoidal dose-response curve calculated EC_50_ with logarithmic and nonlogarithmic concentrations for ABTS and DPPH free radicals.

	DPPH	ABTS
EC_50_	57.19 *μ*g/mL (95% CI: 46.06–71.20)	31.5 *μ*g/mL (95% CI: 26.71–37.16)
Log EC_50_	1.757 *μ*g/mL (95% CI: 1.663–1.852)	1.498 *μ*g/mL (95% CI: 1.427–1.570)
Hill slope	0.6666	0.7235
*R*-squared (*R*^2^)	0.9384	0.965

Abbreviations: EC_50_: the half-maximal effective concentration, log: logarithm, DPPH: 2,2-diphenyl-1-picrylhydrazyl, ABTS: 2, 2′-azino-bis (3-ethylbenzothiazoline-6-sulfonic acid), and CI: confidence interval.

**Table 6:** The MIC and MBC of ZnO-NPs against microorganisms.

Microorganism	MIC (*μ*g/mL)	MBC (*μ*g/mL)
*E. coli*	61.30	312
*B. subtilis*	14.50	G
*A. baumannii*	86.21	G
*S. aureus*	0.098	156
*P. aeruginosa*	206.1	G
*C. albicans*	14.46	G

Abbreviations: G: growth, MIC: minimum inhibitory concentration, and MBC: minimum bactericidal concentration.

## Data Availability

The data associated with this study can be accessed from the first author upon a reasonable request.
